# Simultaneous Gastric Cancer Metastases to the Small and Large Intestines: Hidden Small Intestinal Lesions and Colonic-Mimicking Metastases

**DOI:** 10.70352/scrj.cr.25-0158

**Published:** 2025-10-28

**Authors:** Sota Nakamura, Manabu Yamamoto, Tsukasa Nakamura, Yuki Tateishi, Ryo Sakada, Shoichiro Nagashima, Kazutoyo Morita, Hidefumi Higashi, Tomoharu Yoshizumi

**Affiliations:** 1 Department of Surgery and Science, Graduate School of Medical Sciences, Kyushu University, Fukuoka, Fukuoka, Japan; 2 Department of Surgery, Fukuoka City Hospital, Fukuoka, Fukuoka, Japan; 3 Department of Hepatology, Fukuoka City Hospital, Fukuoka, Fukuoka, Japan; 4 Department of Anatomic Pathology, Pathological Sciences, Graduate School of Medical Sciences, Kyushu University, Fukuoka, Fukuoka, Japan

**Keywords:** gastric cancer, simultaneous metastases, small intestine, large intestine

## Abstract

**INTRODUCTION:**

Gastric cancer often presents with metastases at diagnosis, but simultaneous metastases to both the small and large intestines are extremely rare and may be misinterpreted as synchronous primary intestinal cancers, particularly when preoperative imaging is inconclusive.

**CASE PRESENTATION:**

A 78-year-old male receiving cabozantinib for hepatocellular carcinoma with vertebral metastasis presented with anorexia, epigastric discomfort, and melena. Endoscopy revealed an ulcerative gastric lesion, and colonoscopy showed irregular ulcerative lesions in the ascending and transverse colons. The patient underwent laparoscopic distal gastrectomy, right hemicolectomy. During surgery, a small intestinal tumor was suspected, prompting an additional partial resection. Histopathology and immunohistochemistry (CK7, CK20, CDX2, SATB2, Arginase-1) confirmed that the intestinal lesions were metastases from gastric cancer rather than synchronous primary colorectal cancers.

**CONCLUSIONS:**

This case suggests that preoperative and intraoperative imaging may not detect rare metastatic patterns, and that immunohistochemical analysis may help estimate tumor origin. Careful differentiation between true synchronous colorectal cancer and gastric cancer with intestinal metastases may help guide treatment decisions.

## Abbreviations

CA19-9
: carbohydrate antigen 19-9
CDX2
: caudal-type homeobox 2
CEA
: carcinoembryonic antigen
CK
: cytokeratin
HCC
: hepatocellular carcinoma
MSI
: microsatellite instability
SATB2
: special AT-rich sequence-binding protein 2

## INTRODUCTION

Gastric cancer is the 5th most common malignancy worldwide in terms of both incidence and mortality.^1)^ At diagnosis, it may present with either local progression or distant metastases,^2)^ most often involving the lymph nodes, liver, bone, lungs, and brain.^3)^ Involvement of the small or large intestines is extremely rare.^4,5)^ Autopsy studies have shown intestinal metastasis in only 3% of large intestinal and 2% of small intestinal cases of gastric cancer.^6)^ Metastatic tumors of the large intestine account for 0.1%–1% of all colorectal malignancies,^7)^ 21.9% of which originate from gastric cancer.^7)^ Most result from peritoneal dissemination or direct invasion, whereas only 3.1% occur via hematogenous or lymphatic spread.^7)^ Similarly, metastatic tumors of the small intestine are uncommon, representing approximately 9% of all malignant small intestinal tumors,^8)^ of which only 2.4%–11.4% are derived from gastric cancer, again usually through peritoneal dissemination or direct invasion.^9,10)^ Hematogenous or lymphatic metastasis to the small intestine alone is extremely rare, with a reported incidence of 2.8%.^10)^ Simultaneous metastasis to both the small and large intestines is exceptionally rare.

By contrast, synchronous double primary cancers of the stomach and colon are reported more frequently, occurring in approximately 4.4% among gastric cancer patients.^11)^ Therefore, when both gastric and colonic tumors are detected simultaneously, they are generally presumed to represent synchronous primary cancers rather than metastatic disease. This diagnostic assumption can make it difficult to identify the rare cases of intestinal metastasis from gastric cancer preoperatively.

Herein, we present a case of gastric cancer with simultaneous metastases to the small intestine and 3 distinct sites in the large intestine, identified during treatment for HCC, and review the relevant literature.

## CASE PRESENTATION

A 78-year-old male presented at our hospital with anorexia, epigastric discomfort, and melena. He had a 45-pack-year smoking history but had quit 12 years earlier, and did not consume alcohol. His family history included his father’s death from liver disease and his brother’s death from liver cirrhosis, with no apparent family history of cancer. Before his visit, he had been receiving treatment for recurrent HCC with vertebral metastases for 10 years, including lenvatinib, atezolizumab plus bevacizumab, and transarterial chemoembolization, followed by cabozantinib at presentation.

Laboratory investigations revealed a white blood cell count of 3.8 × 10^6^/L, hemoglobin level of 101 g/L, total protein level of 63 g/L, and albumin level of 32 g/L. Serum CEA (2.1 ng/mL) and CA19-9 (4 U/mL) were within normal limits.

**Fig. 1:**
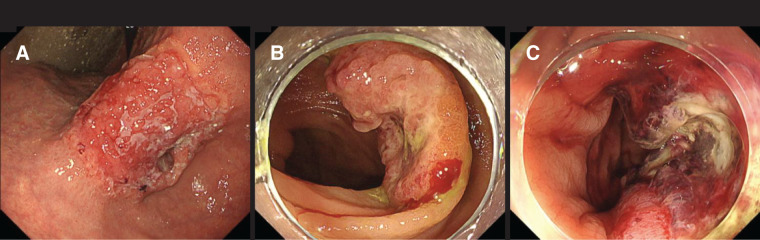
Findings of upper gastrointestinal endoscopy. (**A**) An ulcerative lesion with raised margins at the lesser curvature extending from the antrum to the body of the stomach. (**B**) Irregularly shaped ulcerative lesion in the proximal ascending colon. (**C**) Irregularly shaped ulcerative lesion in the transverse colon.

Esophagogastroduodenoscopy showed an ulcerative lesion with raised margins on the lesser curvature, extending from the antrum to the body of the stomach. The pre-pyloric region exhibited circumferential stenosis, nearly occluding the lumen, contributing to poor oral intake; however, the pyloric ring remained intact (**Fig. 1A**). Biopsy confirmed poorly differentiated adenocarcinoma. Preoperative colonoscopy revealed irregular, ulcerated, elevated lesions with relatively well-defined margins in both the ascending and transverse colons, and biopsies again demonstrated poorly differentiated adenocarcinomas (**Fig. 1B**, **1C**). Because all 3 lesions were poorly differentiated adenocarcinomas, morphological comparison among them was inherently difficult, and as immunohistochemical staining was not performed, evaluation of concordance between the gastric and colonic tumors was limited; nevertheless, their endoscopic appearance was more consistent with primary colon cancers than with metastases.

**Fig. 2:**
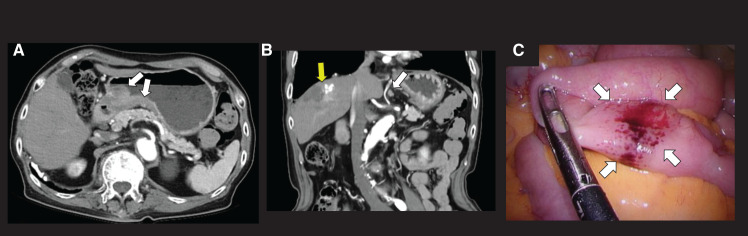
Findings of CT and intraoperative laparoscopic observation. (**A**) Wall thickening with enhancement from the lesser curvature extending from the gastric angle to the antrum (white arrows). (**B**) In the coronal section of the CT, enlarged lymph nodes near the gastric cardia to celiac regions (white arrow). Hepatocellular carcinoma after hepatic arterial chemoembolization (yellow arrow). (**C**) A small intestine tumor was suspected under intraoperative laparoscopic observation.

CT scans showed a thickened gastric antrum wall with enlarged cardiac to celiac lymph nodes but no distant metastases (**Fig. 2A**, **2B**). The colonic lesions were inconspicuous on CT. PET was not performed due to limited accessibility. Given the higher prevalence of synchronous primary gastric and colorectal cancers compared with synchronous gastric metastases to the colon, the preoperative diagnosis was primary gastric cancer with synchronous double colon cancers.

The patient developed progressive anemia with hemoglobin declining to the 8 g/dL range, likely due to chronic oozing from gastric and colonic lesions, necessitating preoperative transfusion. Combined poor oral intake from gastric outlet obstruction and a controlled HCC with estimated life expectancy exceeding 1 year, surgical intervention was deemed appropriate. Curative resection was planned instead of palliative surgery. Accordingly, the patient underwent laparoscopic distal gastrectomy and right hemicolectomy.

Intraoperatively, thorough laparoscopic inspection of the liver surface, abdominal wall, small bowel mesentery, colonic mesentery, and pelvic floor revealed no nodular lesions suggestive of peritoneal dissemination. To further exclude peritoneal involvement, permanent cytology of peritoneal lavage fluid was performed; however, the specimen was inadequate for definitive evaluation. A suspected small intestinal tumor was noted (**Fig. 2C**), which led to additional partial resection of the small intestine.

**Fig. 3:**
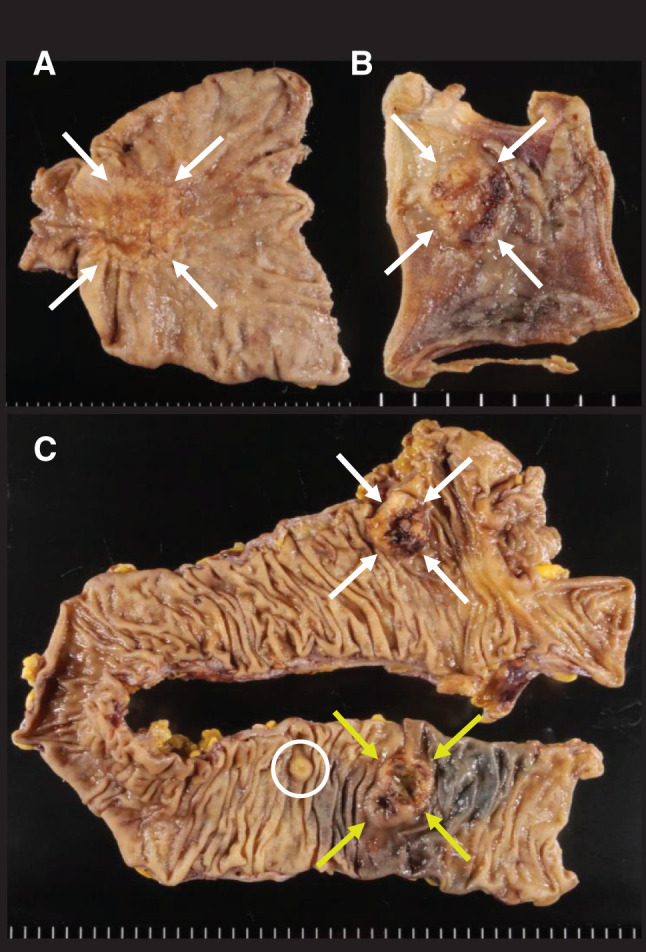
Resected specimens. (**A**) The huge gastric tumor measures 58 × 45 mm from the body to the antrum of the stomach (white arrows). (**B**) The small intestine tumor was a 12 × 10 mm elevated lesion (white arrows). (**C**) The tumor size of the ascending colon lesion was 23 × 20 mm (white arrows). The tumor size of transverse colon lesions were 28 × 20 mm (yellow arrows) and 8 × 8 mm (white circle). All lesions are elevated and ulcerative.

Macroscopically, elevated lesions were observed in the resected specimens: 58 × 45 mm in the stomach (**Fig. 3A**), 12 × 10 mm in the small intestine (**Fig. 3B**), 23 × 20 mm in the ascending colon, and 2 in the transverse colon (28 × 20 mm and 8 × 8 mm; **Fig. 3C**). The smaller transverse colonic lesion (8 × 8 mm) was incidentally identified postoperatively.

**Fig. 4:**
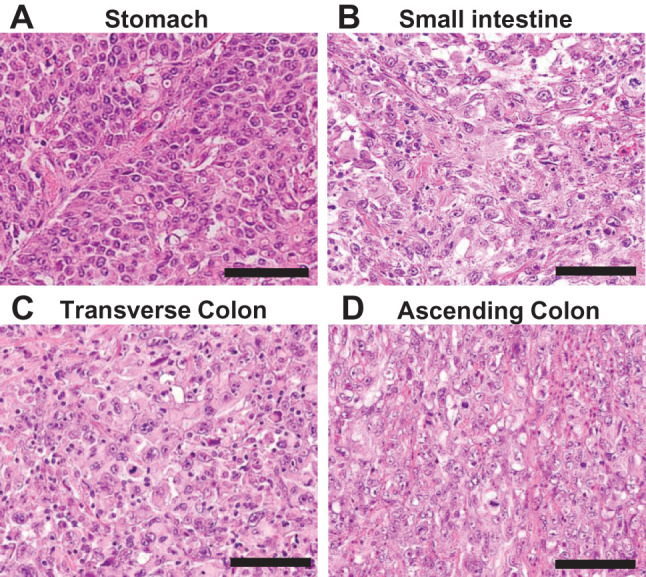
Histological findings. (**A**) The gastric lesion was a poorly differentiated adenocarcinoma (H&E, ×200). Scale bar, 100 μm. Both (**B**, **C**) the large intestinal lesions and (**D**) the small intestinal lesion are poorly differentiated carcinomas resembling the gastric lesion (H&E, ×200). Scale bar, 100 μm. H&E, hematoxylin and eosin

Histopathological examination with hematoxylin and eosin staining revealed that the gastric lesion was a poorly differentiated adenocarcinoma (**Fig. 4A**), invading the serosa (T4a), with lymph node metastases (19 out of 29 nodes positive, located in the cardiac-to-celiac region), extranodal extension, and perineural invasion. Surgical margins were clear. The small and large intestinal lesions exhibited similar histological features and were also diagnosed as poorly differentiated adenocarcinomas (**Fig. 4B**–**4D**). Morphologically, the intestinal tumors were predominantly submucosal, expanding to compress both mucosal and deeper layers, with the smallest colonic lesion confined to the mucosa and submucosa.

**Fig. 5:**
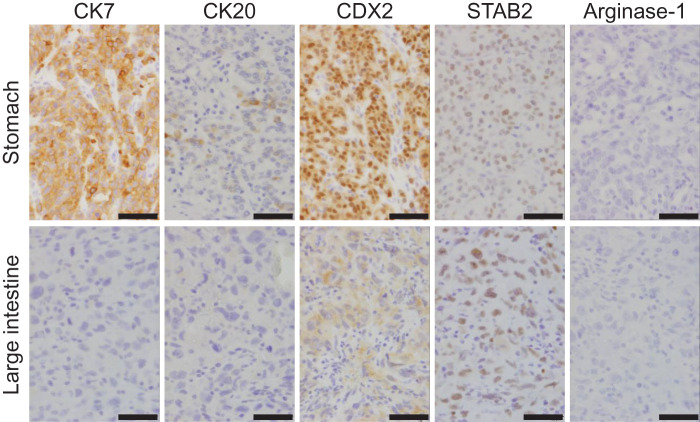
Representative immunohistochemical staining of gastric and colonic lesions. The gastric lesion showed positivity for CK7, focal CK20, diffuse CDX2, and weak SATB2, consistent with primary gastric adenocarcinoma. The colonic lesion was negative for CK7, CK20, SATB2, and Arginase-1, with only weak CDX2 expression. Arginase-1 was negative in both gastric and colonic lesions, excluding the possibility of hepatocellular carcinoma metastasis. Scale bar, 40 μm.

Immunohistochemically, the gastric tumor cells were positive for CK7, showed very focal CK20, with focal and weak SATB2, and diffuse CDX2 expression, consistent with primary gastric adenocarcinoma. By contrast, the 3 intestinal lesions were negative for CK7 and CK20, with CDX2 expression ranging from negative to weakly positive. Representative immunostaining of the gastric tumor and 1 colonic lesion is shown in **Fig. 5**, including CK7, CK20, CDX2, SATB2, and Arginase-1. Hepatocyte, Arginase-1, and Glypican-3 staining of this colonic lesion was negative, excluding metastatic hepatocellular carcinoma. For completeness, Arginase-1 staining was also negative in the remaining 2 intestinal lesions and the gastric tumor, confirming the absence of HCC metastasis in all tumors.

Based on the immunohistochemical profile and the submucosal-predominant growth pattern of the intestinal lesions with compressive extension into adjacent layers, the final diagnosis was primary gastric carcinoma with metastases to the small and large intestines.

The patient recovered without any obvious complications and was discharged on POD 19. However, contrast-enhanced CT on POD 26 revealed multiple lymph node metastases, including the right axillary, tracheobronchial, and right hilar lymph nodes. MSI testing of the gastric tumor revealed MSI-high status. Pembrolizumab was therefore proposed for the treatment of recurrent MSI-high gastric cancer; however, the patient declined further therapy and received palliative care. The patient ultimately died of myocardial infarction 3 months postoperatively.

## DISCUSSION

Primary metastatic pathways of gastric cancer include hematogenous spread, lymphatic spread, direct invasion, and peritoneal dissemination.^12)^ Metastases to both the small and large intestines without peritoneal dissemination or direct invasion are exceedingly rare.^7,13)^ Distinguishing synchronous primary colorectal cancers from intestinal metastases can be challenging, particularly when preoperative imaging does not suggest direct invasion or peritoneal dissemination.^14)^

In the present case, preoperative colonoscopy revealed irregular ulcerative, elevated lesions in the ascending and transverse colon. Biopsies from these lesions and the gastric tumor all showed poorly differentiated adenocarcinoma. Metastatic intestinal tumors typically present with submucosal tumor-like morphology,^14)^ whereas the endoscopic appearance here resembled primary colorectal cancer. Epidemiologically, synchronous primary colorectal cancers are more common than simultaneous intestinal metastases from gastric cancer, which initially led to misinterpretation of the colonic lesions.

Postoperative histopathological examination demonstrated that the intestinal lesions were metastases from the primary gastric cancer. They were predominantly submucosal with compressive extension into adjacent layers, a pattern consistent with secondary origin. Typically, primary colorectal adenocarcinomas are CK7-negative and strongly express CK20, CDX2, and SATB2.^15,16)^ In our case, the intestinal tumors were negative for CK7 and CK20, with absent to weak CDX2 expression, and only weak SATB2 staining. This immunoprofile differs from that of primary colorectal cancer, which generally shows strong CDX2 and SATB2 positivity. By contrast, metastatic gastric cancer may display variable CK7, CK 20, and CDX2 expression.^17,18)^ Although the gastric tumor was CK7-positive and the intestinal lesions were CK7-negative, previous studies have reported such discrepancies between primary gastric cancers and their metastases.^19)^ Additional immunohistochemical staining (Hepatocyte, Arginase-1, and Glypican-3) was negative, effectively excluding hepatocellular carcinoma as the origin. Taken together, these pathological and immunohistochemical findings strongly indicate that the intestinal lesions represented metastases from gastric cancer rather than synchronous primary colorectal cancers.

The distribution and morphology of the metastatic lesions suggest lymphatic dissemination via the mesenteric route. This is supported by several observations: (1) multiple lymph node metastases were present around the celiac and superior mesenteric arteries, (2) metastatic lesions were mainly located in the submucosa and muscularis propria along the mesenteric pathway, and (3) although uncommon, synchronous intestinal metastases can occur in gastric cancers with extensive lymphovascular invasion.^20)^ While hematogenous spread cannot be completely ruled out, the morphology and distribution pattern are more consistent with lymphatic dissemination.

Reports of colonic metastases from gastric cancer note that multiple synchronous lesions usually appear as polypoid or flat-elevated lesions.^14)^ To our knowledge, no cases have been reported in which multiple colonic lesions presented as ulcerated elevated tumors (type 2 lesions) initially suspected to be primary colorectal cancers.^14)^ Colonic metastases of gastric cancer via lymphatic spread can closely mimic the morphology of primary colorectal cancer,^14)^ making it particularly difficult to distinguish them from synchronous colorectal cancer when multiple lesions are present.

Although laparoscopy was used to assess the abdominal cavity, the limited field of view prevents absolute exclusion of microscopic peritoneal dissemination. Similarly, PET/CT was not performed due to limited accessibility, potentially further restricting preoperative detection of small or occult metastases. Pathological examination, however, confirmed that the 3 colonic and 1 small intestinal metastatic lesions did not involve the serosal surface, making peritoneal dissemination an unlikely metastatic pathway.

The patient had received long-term chemotherapy for HCC, which might have influenced tumor biology and metastatic patterns, although direct evidence linking HCC treatment to altered gastric cancer metastasis is lacking. Previous studies have suggested that preoperative chemotherapy can modify metastatic behavior in gastric cancer,^21)^ and some agents, such as cabozantinib, have been reported to potentially suppress peritoneal dissemination.^22)^ These findings raise the unproven possibility that prior chemotherapy for HCC could have affected the metastatic pattern observed in this case. Further studies are needed to clarify these potential effects.

Surgical intervention was performed due to progressive anemia, gastric outlet obstruction, and the patient’s controlled HCC with an expected survival exceeding 1 year. Curative resection was chosen over palliative measures. If the metastatic nature of these lesions had been recognized preoperatively, systemic chemotherapy or bypass surgery combined with chemotherapy might have been more appropriate, as previous studies report improved symptom control and survival with these approaches in advanced gastric cancer.^2,12,23)^ This case demonstrates that distinguishing true synchronous colorectal cancer from gastric cancer with synchronous intestinal metastases is crucial for guiding treatment decisions and cannot rely solely on probabilistic assumptions.

Several lessons emerge from this case. First, gastric cancer metastases to the intestine can mimic primary intestinal cancers on endoscopy and imaging, particularly when lymphatic spread is suspected. Second, immunohistochemical profiling may help determine tumor origin and could be performed early when multiple suspicious lesions are present.

Whether small intestinal metastases should be routinely investigated preoperatively in patients with advanced gastric cancer remains controversial.^4)^ Owing to their extreme rarity, no large-scale analyses are available. A review of prior cases of small intestinal metastasis from gastric cancer (excluding direct invasion or peritoneal dissemination) identified only 6 reports from 1950 to 2025 (**Table 1**).^5,13,24–27)^

**Table 1:** Previous reports of metastases to the small intestine of gastric cancer

No.	Author	Year	Age (y)	Sex	Histotype of gastric cancer	Metastasis location	Synchronous/Metachronous	Mechanism of metastasis	Clinical presentation	CT findings of small intestine	Methods for detecting	Treatment of metastasis	Survival (weeks)
1	Choudhry et al.^24)^	2009	77	M	tub2	Ileum	Metachronous	ND	Intestinal obstruction	Negative	Intraoperative	Partial resection	ND
2	Kamiyama et al.^5)^	2014	70	M	por > tub, sig	Ileum	Synchronous	Lymphatic	Anorexia, weight loss	Positive	Intraoperative	Partial resection	13
3	Urakawa et al.^13)^	2017	67	M	tub2	Jejunum	Metachronous	Hematogenous	Perforation	Negative	Postoperative pathological examination	Partial resection	15
4	Nishimura et al.^25)^	2018	72	M	ND	Ileum	ND	ND	Intestinal obstruction	Negative	VCE, DBE	Partial resection	13
5	Fan and Su^26)^	2019	64	F	por > sarcoma	Jejunum	Synchronous	Hematogenous	Melena	Negative	PET-CT	Partial resection	6
6	Kubota et al.^27)^	2020	76	M	por	ND (multiple)	Metachronous	ND	Fecal occurt blood positive	ND	Enteroscopy	Chemotherapy	72
7	Our case	2024	78	M	por	Jejunoileum	Synchronous	Lymphatic suspected	Anorexia, melena	Negative	Intraoperative	Partial resection	9

DBE, double-baloon endoscopy; ND, not described; por, poorly differentiated adenocarcinoma; sig, signet ring cell carcinoma; tub2, moderately differentiated tubular adenocarcinoma; VCE, video capsle endoscopy

Most reported lesions occurred in males, were poorly differentiated, and presented with complications such as bowel obstruction, bleeding, or perforation, for which partial resection was usually performed. Notably, small intestinal metastasis was detected preoperatively in only 3 of 7 cases, in which advanced modalities such as PET-CT, enteroscopy, video capsule endoscopy, or double-balloon endoscopy were employed. In more than half of the reported cases, diagnosis was made intraoperatively or postoperatively, indicating the inherent limitations of routine preoperative imaging studies.

In cases where synchronous colonic metastases from gastric cancer are suspected, as in the present patient, preoperative evaluation of the small intestine may be considered, since additional lesions could be missed by routine imaging.

## CONCLUSIONS

Simultaneous metastases of gastric cancer to both the small and large intestines are extremely rare and can mimic synchronous primary colorectal cancers. Immunohistochemical analysis may help estimate tumor origin in such cases. Awareness of this mimicking potential may support appropriate preoperative evaluation and treatment planning.

## DECLARATIONS

### Availability of data and materials

All related data are included within the article.
